# Magnetically Actuated Biodegradable Nanorobots for Active Immunotherapy

**DOI:** 10.1002/advs.202300540

**Published:** 2023-06-29

**Authors:** Yicheng Ye, Hao Tian, Jiamiao Jiang, Weichang Huang, Ruotian Zhang, Huaan Li, Lu Liu, Junbin Gao, Haixin Tan, Meihuan Liu, Fei Peng, Yingfeng Tu

**Affiliations:** ^1^ NMPA Key Laboratory for Research and Evaluation of Drug Metabolism & Guangdong Provincial Key Laboratory of New Drug Screening School of Pharmaceutical Sciences Southern Medical University Guangzhou 510515 China; ^2^ School of Materials Science and Engineering Sun Yat‐Sen University Guangzhou 510275 China

**Keywords:** antigen cross‐presentation, biodegradable robots, immunotherapy, magnetic actuation, melanoma

## Abstract

An efficient and cost‐effective therapeutic vaccine is highly desirable for the prevention and treatment of cancer, which helps to strengthen the immune system and activate the T cell immune response. However, initiating such an adaptive immune response efficiently remains challenging, especially the deficient antigen presentation by dendritic cells (DCs) in the immunosuppressive tumor microenvironment. Herein, an efficient and dynamic antigen delivery system based on the magnetically actuated OVA‐CaCO_3_‐SPIO robots (OCS‐robots) is rationally designed for active immunotherapy. Taking advantage of the unique dynamic features, the developed OCS‐robots achieve controllable motion capability under the rotating magnetic field. Specifically, with the active motion, the acid‐responsiveness of OCS‐robots is beneficial for the tumor acidity attenuating and lysosome escape as well as the subsequent antigen cross‐presentation of DCs. Furthermore, the dynamic OCS‐robots boost the crosstalk between the DCs and antigens, which displays prominent tumor immunotherapy effect on melanoma through cytotoxic T lymphocytes (CTLs). Such a strategy of dynamic vaccine delivery system enables the active activation of immune system based on the magnetically actuated OCS‐robots, which presents a plausible paradigm for incredibly efficient cancer immunotherapy by designing multifunctional and novel robot platforms in the future.

## Graphical abstract

Magnetically actuated biodegradable CaCO_3_‐based nanorobots are able to improve the crosstalk between antigens and DCs, followed by efficient lysosome escape and antigen cross‐presentation of DCs. This work presents a facial and multifunctional smart robot platform for efficient cancer therapy with immunomodulation‐enhanced antitumor immunity, which represents an outstanding therapeutic strategy for tumor immunotherapy.

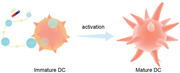

## 1 Introduction

Cancer is still a major killer threatening human health, with the mortality rate rising recently and causing a heavy economic burden simultaneously.^[^ ^1^, ^2^ ^]^ Conventional clinical cancer treatments normally include surgery, radiotherapy, and chemotherapy, which are often disappointed with either high risk of tumor recurrence or severe systemic side effects.^[^ ^3^ ^]^ Cancer immunotherapy can regulate the immune system from the host to combat with cancer cells, which has attracted tremendous attention in recent years.^[^ ^4^, ^5^ ^]^ Normally efficiently processing antigens by antigen‐presenting cells (APCs) is the first step to initiate the immune response.^[^ ^6^ ^]^ Humoral response and cellular response are both components of antigen‐induced specific immunity responses. In general, humoral response is triggered when exogenous antigens are presented by MHC II molecules to CD4^+^ T lymphocytes and the cellular response is normally activated when endogenous antigens are presented by MHC I molecules to CD8^+^ T lymphocytes.^[^ ^7^ ^]^ For the acidic tumor microenvironment, several strategies of stimulating responsiveness were employed to activate the immune response and suppress the tumor.^[^ ^8^, ^9^, ^10^, ^11^ ^]^ However, due to inactive APCs in the immunosuppressive tumor microenvironment, the release of tumor antigens namely endogenous antigens are not sufficient to initiate the immune system.^[^ ^12^ ^]^ Moreover, the exogenous antigens from vaccines captured by APCs are typically internalized in the lysosomes which was presented by MHC II pathway to produce humoral immunity response rather than satisfactory cellular immunity response.^[^ ^13^, ^14^, ^15^ ^]^ Therefore, rational design of antigens delivery system with immunomodulatory properties and efficient antigens cross‐presentation through MHC I pathway is highly desirable to initiate the powerful cellular immunity for antitumor therapy.

Recently, various strategies have been elaborately designed and employed to realize lysosome escape and facilitate antigen cross‐presentation for vaccines delivery.^[^ ^16^, ^17^, ^18^ ^]^ For instance, “proton sponge” effect enables that wrapping the vaccine delivery systems with positively charged polymers or peptides can promote their escape from lysosome.^[^ ^19^, ^20^ ^]^ Nevertheless, the transition from the bench to the clinic will be significantly hindered by the demanding fabrication approaches, challenging modification strategies and biosafety worries regarding the extraneous components. Moreover, these encouraging lysosome escape strategies mainly rely on the passive diffusion of the carrier systems, which lack of sufficient impetus to pass through the compact tissue barriers as well as the active targeting delivery, leading to the compromised effectiveness of the vaccine.

Micro‐/nano‐robots are miniaturized machines or devices that are capable of transforming diverse energies from the surrounding environment into autonomous motion in fluid, driven by either chemical reactions (catalytic^[^ ^21^, ^22^, ^23^ ^]^ or enzymatic reactions^[^ ^24^, ^25^, ^26^ ^]^) or external physical fields (light,^[^ ^27^, ^28^, ^29^ ^]^ ultrasound,^[^ ^30^, ^31^, ^32^ ^]^ magnetic,^[^ ^33^, ^34^, ^35^, ^36^, ^37^ ^]^ and electrical field^[^ ^38^, ^39^, ^40^ ^]^) or microorganisms such as sperm cells^[^ ^41^, ^42^ ^]^ or bacteria^[^ ^43^ ^]^ or a combination of the above.^[^ ^44^, ^45^ ^]^ Benefiting from the mobility at a tiny scale and strong cargo towing, micro‐/nano‐robots show limitless potential to revolutionize various research fields. In particular, magnetically actuated micro‐/nano‐robots have drawn significant attentions due to their wireless and precise motion control as well as high penetrating capacity.^[^ ^46^, ^47^ ^]^ Magnetic micro‐/nano‐robots with adjustable motion capabilities are able to be navigated into hard‐to‐reach position, enabling a wide range of biomedical applications, such as single cell surgery,^[^ ^48^ ^]^ precise neuronal differentiation,^[^ ^49^ ^]^ thrombus removal,^[^ ^50^ ^]^ active drug delivery,^[^ ^35^ ^]^ and directing the T cells chemotaxis.^[^ ^51^ ^]^ However, utilizing magnetic micro‐/nano‐robots for the activation of immune system has been rarely reported. Recently, Dong et al. demonstrated the ultrasound‐propelled gold nanowires wrapped by OVA for enhanced cellular immunity.^[^ ^52^ ^]^ With the remarkable ability of active movement, the ultrasound‐propelled nanorobot exhibited prominent advantages as a novel vaccine delivery system compared to the static one. However, multifunctional micro‐/nano‐robots with excellent biosafety and flexibility are highly expected to move forward in clinical field.

**Figure 1:**
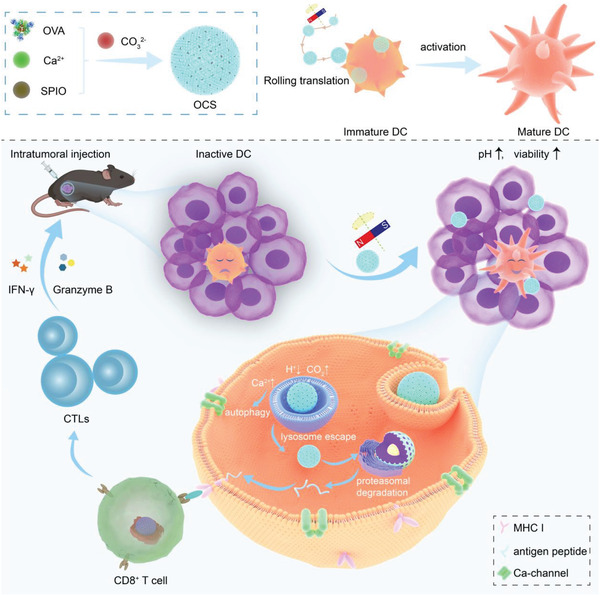
Schematic illustration of the fabrication of OCS‐robot and its application for activating the immune system against melanoma. The OCS‐robots were intratumorally injected into a B16‐OVA tumor‐bearing mouse. When applying the rotational magnetic field, the OCS‐robots achieved active motion to attenuate the acidic tumor microenvironment and recover the viability of DCs. Thanks to the active motion, the OCS‐robots were capable to target the infiltrated DCs and enhance the endocytosis process, followed by lysosome escape and antigen cross‐presentation, thus initiating the cellular immunity for cancer therapy in vivo.

Here, we present the study of magnetically actuated biodegradable OCS‐robot for enhanced antigen cross‐presentation and active melanoma immunotherapy in vivo (**Figure** **1**). The OCS was synthesized based on biomimetic strategy in which OVA antigens were employed as templates. The integration of SPIO enabled the wireless actuation and manipulation of OCS‐robots under the rotational magnetic field. On the one hand, as the DCs are inactive in the acidic tumor microenvironment, the developed OCS‐robots were able to be navigated to the tumor region and neutralize the tumor microenvironmental acidity to recover the viability of DCs. On the other hand, with the active motion ability of OCS‐robots, the contact and uptake efficiency by DCs were increased accordingly, followed by the efficient lysosome escape and antigen cross‐presentation, thus triggering the cellular immunity for antitumor therapy in vivo. Such an OCS‐robot system is capable of activating the immune system from the host to fight tumors without additional drugs, which represents a new generation of dynamic vaccine system and advances the efficiency of active and noninvasive tumor immunotherapy.

## 2 Results and Discussion

### 2.1 Fabrication and Characterization of OCS‐Robot

**Figure 2:**
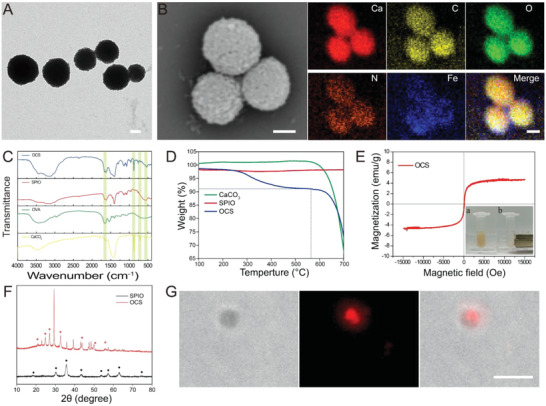
Characterization of OCS. A) TEM image of OCS. Scale bars, 200 nm. B) SEM image of OCS and the corresponding EDX mapping for Ca, C, O, N, and Fe respectively. Scale bars, 300 nm. C) FTIR spectra of pristine CaCO_3_, OVA, SPIO, and OCS respectively. D) Thermo‐gravimetric (TG) analysis of pristine CaCO_3_, SPIO, and OCS respectively. E) Magnetic hysteresis loops of OCS. The inserted picture represented the magnetic response capacity. F) XRD patterns of SPIO and OCS. G) Bright field image and the corresponding fluorescence image of OCS. Scale bars, 5 µm.

In order to actively activate the immune response, magnetically actuated OCS nanorobots were designed and fabricated to dynamically deliver the antigens OVA to the APCs. Inspired by the biomineralized construction strategy, these microrobots with high biocompatibility and biodegradability were constructed by a one‐pot synthesis.^[^ ^53^ ^]^ The as synthesized superparamagnetic iron oxide (SPIO) was spherical and uniform with a size distribution of 11 nm (Figure S1, Supporting Information) according to TEM statistical analysis and 50 nm from DLS measurement (Figure S2, Supporting Information). Based on the templating effect of antigen OVA, the spherical CaCO_3_ particles were successfully synthesized with SPIO loading inside. As shown in **Figure** **2**A, the uniform in size of OVA‐CaCO_3_‐SPIO (OCS) with hierarchical clusters around was clearly observed from the TEM image, which presented the spherical morphology with an average diameter of 500 nm. It was also determined that the obtained OCS particles were around 600 nm in size by DLS (Figure S2, Supporting Information). Furthermore, the honeycomb and porous structure was clearly displayed in the SEM image (Figure 2B), which was favorable for the acidic responsive release of OVA (Figure S3, Supporting Information). The corresponding energy‐dispersive X‐ray spectroscopy (EDX) mapping of Ca (16.74%), C (19.08%), O (55.71%), N (6.09%), and Fe (2.38%) were presented respectively (Figure S4, Supporting Information), indicating the existence of OVA and SPIO in the OCS robot. Accordingly, the zeta potential of OCS changed from 36.4 mV (SPIO) to −19.2 mV (Figure S5, Supporting Information). Besides, OVA and SPIO were further confirmed by Fourier transform infrared spectra (Figure 2C). The characteristic peak (CO_3_^2−^, 724 and 876 cm^−1^) was assigned to the in‐plane and out‐of‐plane deformation vibration peak of CaCO_3_, with the characteristic peak (C=O, 1650 cm^−1^) and (Fe—O, 532 cm^−1^) of OVA and SPIO respectively, confirming the successful encapsulation of OVA and SPIO in robot again. In order to determine the loading capacity of antigen OVA, thermo‐gravimetric analysis was then employed. As shown in Figure 2D, the weight loss at 200–560 °C was assigned to the content of OVA in the OCS, while the weight loss at 560–700 °C was due to the thermal decomposition of CaCO_3_, with no change of SPIO. Therefore, the loading capacity of OVA antigen was calculated to be 7.6% in weight. Due to the entrapped SPIO, the magnetic hysteresis loops showed that OCS robot was superparamagnetic, and the saturation magnetization was around 6 emu g^−1^ (Figure 2E). The inserted picture in Figure 2E demonstrated that an external magnet was sufficient to induce the motion of OCS to one side, which showed that OCS robot had good magnetic responsiveness. The porous structure of OCS encouraged us to determine the crystal type, which showed in the vaterite crystallization by the X‐ray diffraction (Figure 2F). The vaterite crystal of CaCO_3_ with porous structure was more sensitive to acidity, which was favorable for attenuating acidity in tumor microenvironment. As demonstrated in Figure S6, Supporting Information, OCS exhibited obvious degradation behavior and gradually became smaller in acidic buffer solution (pH 6.5 and pH 5.5). Besides, the red fluorescence of Cy5 was merged well in the position of OCS in the bright field image (Figure 2G), allowing for the tracking in inverted fluorescence microscope and in vivo imaging (Figure S7, Supporting Information). In view of the presence of SPIO, the enhancement of *T*_2_ signal was also investigated by MRI imaging. It was clearly to see a darkening effect of concentration‐gradient dependence in Figure S8, Supporting Information. The corresponding relaxation (*r*_2_) was determined to be 9.37 mg^−1^ mL s^−1^, which was obtained by plotting the slope of 1/*T*_2_ signal with respect to OCS concentration. The above‐mentioned results confirmed that OCS robot was successfully fabricated, thus providing a foundation for the activation of immune system.

### 2.2 Controllable Locomotion of Magnetically Actuated OCS‐Robots

**Figure 3:**
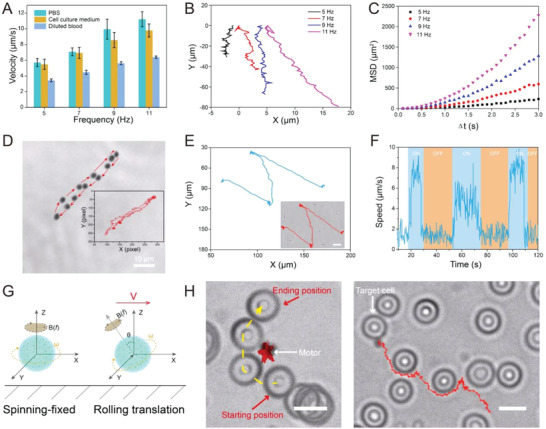
Controllable motion of magnetically actuated OCS‐robot. A) Velocity of OCS‐robot under rotating magnetic field with different frequencies in various biological fluids. Data are represented as the mean ± SD (*n* = 5). B) Representative trajectories of magnetically actuated OCS‐robot under the rotating magnetic field with different frequencies. C) Parabola fitting to plots of mean squared displacement (MSD) obtained from the optical tracking. D) Time‐lapse image and the relevant trajectories showing controllable magnetic propulsion of OCS‐robot in the advance and return moving behavior. E) Trajectories and video snapshot of OCS‐robot performing a predefined track of “N” and the F) corresponding “On–Off” motion speed versus time profile. G) Schematic illustration of magnetic actuation of OCS‐robot, wherein V denotes the translational velocity, B indicates the strength of the magnetic field, f denotes the input frequency of the magnetic field, *ω* indicates the rotation velocity of OCS‐robot, and *θ* represents the tilt angle between OCS‐robot's rotation axis and *z* axis. H) OCS‐robot transporting a single cell in the spinning‐fixed motion behavior and targeting a specific cell in the rolling translation motion behavior. Scale bar, 10 µm.

Upon successful fabrication of OCS robot, the motion behavior was then systematically investigated. Benefiting from the superior magnetics responsiveness of SPIO in OCS, the motion of robot could be actuated in the magnetic field. Therefore, rotating magnetic field generated by a three‐orthogonal Helmholtz coil pair system was carried out to control the movement of our OCS robot. The motion capability of robot in various biological fluids, including PBS solution, cell culture medium, and diluted whole blood were then evaluated. The relationship between the average velocity of robot in different biological fluids and input rotating frequency of the external magnetic field was illustrated in **Figure** **3**A, while altering the magnetic field strength had little impact on the speed of robots compared to the magnetic field frequency (Figure S9, Supporting Information). The average speed of robot increased proportionally with the input frequency within a certain range in the same fluid (Movie S1, Supporting Information). Furthermore, the average speed of robot was maximum in PBS solution (11.2 µm s^−1^) and minimum in diluted whole blood (6.4 µm s^−1^), owing to the different viscosity and composition. The robots were capable to be actuated in various fluids, holding great promise for in vivo biomedical application. The representative tracking trajectories of robot at various input frequency of the external magnetic field in PBS fluids was presented in Figure 3B. It could be seen that the motion of robot was directional and the displacement was longer along with the increased input frequency from trajectories analysis. The corresponding mean square‐displacement (MSD) analysis was displayed in Figure 3C. It was found that the MSD exhibited a parabolic fitting plot, indicating the directional motion of robot. In addition to the directional motion, the robot also exhibited controllable movement and navigation in magnetic field along the predesigned path. It can be clearly found that the robot displayed intelligent reciprocating motion (Figure 3D and Movie S2, Supporting Information) and circular motion behavior (Figure S10 and Movie S3, Supporting Information), performing the task when arriving at a specific location and returning back, which could be navigated to perform specific task into hard‐to‐reach position. Besides, the robot not only exhibited “On–Off” motion control but also achieved controllable navigation along a predetermined pathway “N” (Figure 3E and Movie S4, Supporting Information). The corresponding speed analysis during On–Off operation was provided in Figure 3F. The magnetically actuated navigation of robot was further investigated in the diluted whole blood. As shown in Figure 3G, the robot displayed two kinds of motion behavior: spinning‐fixed and rolling translation. This was because the different input pitch angle even at the same magnetic field. The robot rotated in place at 0° of the input pitch angle of the magnetic field while rolling forward at 90°. Therefore, when the robot is rotating in place, it could deliver one single cell from side to side with the generated mechanical force (Movie S5, Supporting Information). When the robot rolling forward, it could intelligently bypass the obstructive cells and target a specific cell (Figure 3H and Movie S6, Supporting Information). In summary, such multifunctional and intelligent OCS‐robot was capable to be precisely and remotely manipulated, which was a promising candidate for biomedical application.

### 2.3 Effect of OCS‐Robots on DC 2.4 Cells

**Figure 4:**
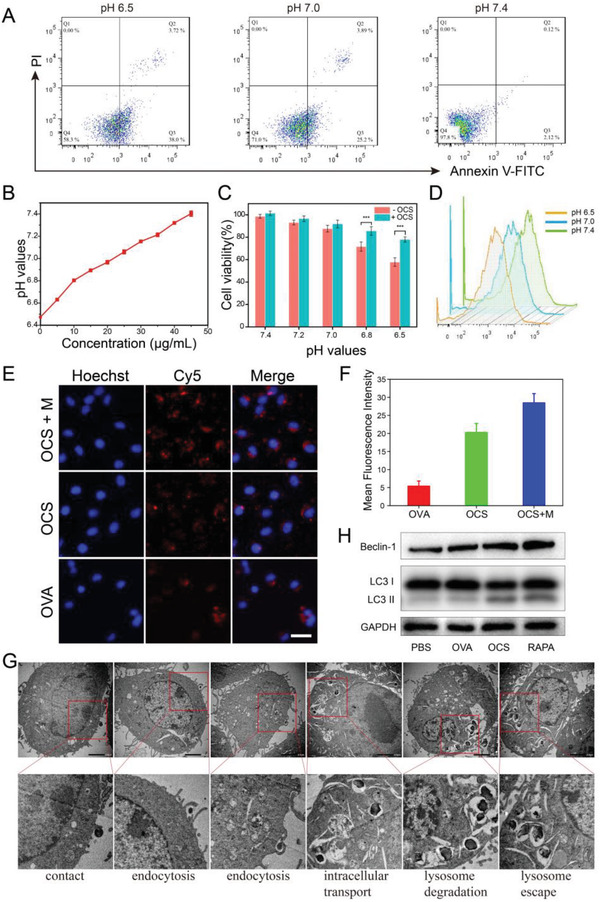
Viability, cellular uptake, and autophagy of DC 2.4 cells after different treatments. A) Apoptotic rates of DC 2.4 cells in various pH values. B) Attenuating the acidity with different concentrations of OCS. Data are represented as the mean ± SD (*n* = 3). C) Viability of DC 2.4 cells under the medium of different pH value with or without OCS for 24 h. Data are represented as the mean ± SD (*n* = 5). Data analyzed by Student's *t*‐tests. *p < 0.05, **p < 0.01, and ***p < 0.001. D) DC 2.4 cells uptake efficiency of OCS in medium of different pH value detected by flow cytometry. E) Intracellular uptake of OCS‐robots at 6 h, and F) the corresponding mean fluorescence intensity analysis. Data are represented as the mean ± SD (*n* = 3). Scale bars, 25 µm. G) Bio‐TEM images of DCs treated with OCS‐robots. Scale bar, 2 µm. H) Western blot analysis of LC 3 and Beclin‐1 protein after different treatments.

DCs, the vital APCs, serve as the inspector to danger signals in the body, processing and presenting the antigen, which is a key prerequisite to mobilize the innate and adaptive immunity of T‐cell initiation. Nevertheless, the viability of DCs is severely inhibited in the acidic tumor microenvironment. Here, lactic acid was employed to regulate the pH value (pH 7.4, 7.0, and 6.5) of the cell culture medium to mimic the acidic tumor microenvironment. After incubating in culture medium with different pH value, DCs were digested and stained with annexin V‐FITC and PI for flow cytometry detection. As depicted in **Figure** **4**A, the percentage of apoptotic cells experienced a massive increase from 2.2% (pH 7.4) to 29.09% (pH 7.0) to 41.72% (pH 6.5), respectively. After adding different concentrations of OCS in the pH 6.5 buffer and incubation for 4 h, the pH value was then detected (Figure 4B). The pH of buffer solution increased with the concentration of OCS and reached 7.4 after adding 45 µg mL^−1^ OCS, indicating OCS‐induced acidity attenuating. Besides, the OCS‐robot could still respond to the external magnetic field when they were degraded partially under acidic microenvironment (Figure S11 and Movie S6, Supporting Information). Meanwhile, the viability of DCs increased from 70% (without OCS) to 85% (with OCS) and 60% (without OCS) to 80% (with OCS) at pH value of 6.8 and 6.5, respectively, which shown significant difference in recovering the viability of DCs (Figure 4C). Accordingly, the viability of DCs also affected the cellular uptake of OCS which could be found by the flow cytometry analysis (Figure 4D) and the corresponding fluorescence intensity was shown in Figure S12, Supporting Information. It was obvious that the efficiency of phagocytosis was inhibited in the acidic microenvironment. Furthermore, the endocytosis of OCS by DCs was also investigated by inverted fluorescence microscope after actively targeting toward the DCs (Figure S13, Supporting Information, and Figure 4E). To determine and optimize the effect of different incubation time on cell phagocytosis, DCs incubated with Cy5 labeled OCS were first treated for 10 min under the magnetic field, followed by different times of incubation. With the active propulsion of OCS‐robot, the red fluorescence signal was observed around the nucleus at 4 h (Figure S14, Supporting Information). When the time of incubation was prolonged to 6 h, the red fluorescence signal of OCS‐robot in the cytoplasm became stronger, with the strongest at 12 h, demonstrating the time‐dependent cellular uptake. For the group of the passive control, after incubation with OVA or OCS at 6 h, both of them displayed a weak red fluorescence signal in the cytoplasm. While red fluorescence signal in the group of OCS was stronger than the group of OVA, it demonstrated that nanoparticles had greater phagocytosis by DCs and longer retention time than free OVA. Whereas, OCS‐robot clearly displayed the strongest fluorescence signal than the control groups, benefitting from the active motion in the magnetic field. It was clearly to see that almost all DCs were surrounded by the red fluorescence at 6 h incubation, demonstrating the suitable incubation time for further research. ImageJ was employed to further evaluate the mean fluorescence intensity. As depicted in Figure 4F, the mean fluorescence intensity of OCS‐robot was 1.4‐fold higher than passive OCS and 5.12‐fold higher than OVA, confirming that OCS‐robot with active and fast propulsion enabled them with distinguished capabilities of cellular penetration. It has been proven that the primary mechanism of robots entering cells is dependent on both clathrin and caveolae‐mediated pathways,^[^ ^54^, ^55^ ^]^ and the active motion of robots increases the probability of contact between the DCs and the antigens, while reducing the required time for contact. Besides, bio‐TEM was also used for the observation of the cellular penetration and transportation process of OCS‐robots (Figure 4G). First, the active motion of OCS‐robots increased the contact between the DCs and the robots, followed by internalization into the DCs by endocytosis. Second, the robots were transported into the lysosome for degradation. Finally, the robots were escaped from the lysosome to the cytoplasm for antigen cross‐presentation due to the “proton sponge effect”. The antigen template assisted particles facilitated the capture by DCs, while the active motion increased the contact between DCs and OCS‐robot. Both of them were beneficial to improve the ability of cellular uptake, which laid out the foundation for the subsequent intracellular vaccine delivery. As autophagy helps to improve the antigen presentation efficiency of DCs,^[^ ^56^ ^]^ whereas the intracellular Ca^2+^ facilitates the occurrence of autophagy,^[^ ^57^ ^]^ the autophagy level of DCs induced by OCS was then investigated. The autophagy associated proteins of LC3 II (transferred from membranous LC3 I) and Beclin‐1 were verified by western blot assays (Figure 4H). OVA control group exhibited a low expression of LC3 II and Beclin‐1, while the expression was upregulated in the OCS group. The OCS served as the antigen delivery carrier, and the Ca^2+^ produced by the OCS encouraged the occurrence of autophagy. All the above results demonstrated OCS‐robot facilitated the recovery of DCs viability and boosted the autophagy level to enhance the antigen presentation.

### 2.4 Lysosomes Colocalization and Escape

**Figure 5:**
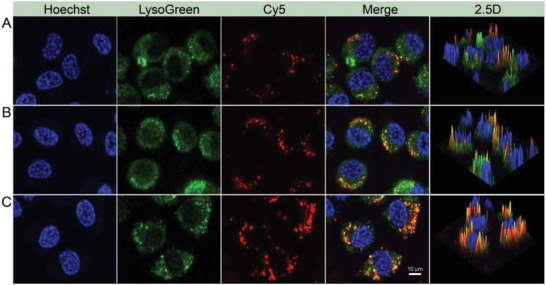
CLSM images of the lysosomes colocalization with different treatments. A) OVA. B) OCS. C) OCS‐robot. The cell nucleus were stained by Hoechst 33342, the lysosomes were stained by LysoTracker green and the OVA/OCS were labeled with Cy5.

Generally speaking, exogenous antigens are uptaken by DCs and internalized into lysosomes for further processing, followed by presenting to CD4^+^ T lymphocytes via MHCII molecules to initiate humoral immunity.^[^ ^6^ ^]^ Therefore, the intracellular fate of OCS was examined to determine its ability to escape from lysosomes and realize the subsequent the cross‐presentation. DCs were first treated with different formulations for 6 h, followed by staining nucleus with Hoechst 33342 and lysosomes with LysoTracker green probe. The colocalization images were acquired by confocal laser scanning microscope (CLSM). As revealed in **Figure** **5**, the red fluorescence was merged well with green fluorescence, indicating that OVA/OCS were first located in lysosomes after uptake by DCs. The image of 2.5D displayed the colocalization results, which demonstrated that the OVA was intracellular distribution rather than extracellular adsorption. Compared with the OVA/OCS group (passive motion), OCS‐robot with active motion in the magnetic field increased the probability of contact with DCs, resulting in enhanced captured ability and the stronger red fluorescence in lysosome (Figure S15, Supporting Information). The ability of OCS‐robot to escape from lysosome was further determined by different time distribution (Figure S16, Supporting Information). The red fluorescence was mostly colocalized with the lysosome after 8 h treatment, while the green fluorescence signal gradually disappeared and the red fluorescence signal turned dispersed after 12 and 16 h treatment. This was because the OCS‐robots were partly dissolved in the acidic lysosomes, disrupting the lysosomes and escaping from them. For the further verification, water‐sac phantom experiment was performed in acetic acid buffer (pH 4.5, lysosome‐mimic medium). The short‐term echo intensity was successively observed in the OCS group, which was attributed to the generation of CO_2_ bubbles by CaCO_3_ in acid (Figure S17, Supporting Information). In contract, there was no echogenicity in the control group. Consequently, a plausible explanation for lysosome escape was related to the “proton sponge effect” and the increased CO_2_ pressure. Antigens escaped from lysosome to the cytoplasm was the key point of cross‐presentation, which promoted the antigen to combine with MHC I molecules and subsequently presented to CD8^+^ T lymphocytes to trigger cellular immunity.

### 2.5 Activation and Maturation of Bone Marrow Dendritic Cells

**Figure 6:**
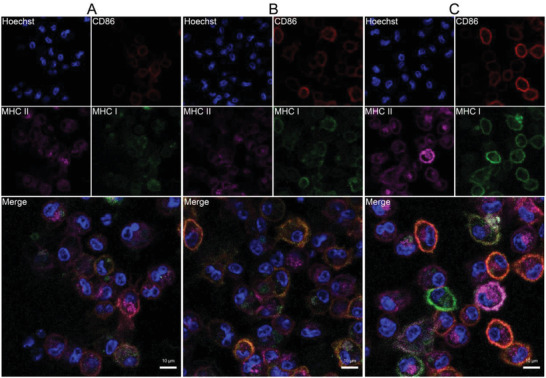
In vivo activation and maturation of BMDCs extracted from mice. A–C) CLSM images of the expression of costimulatory marker CD86, MHC II and MHC I with different treatments. A) OVA. B) OCS. C) OCS‐robots.

Activation and maturation of DCs is a vital step of generating effective immune responses, accompanied by the upregulation expression of costimulatory and MHC molecules. As depicted in Figure S18, Supporting Information, the activation and maturation of DCs (CD80^+^CD86^+^) after OCS‐robots treatment (48.1%) was significant higher than that of PBS control (25.7%), which was ascribed to both active motion and intrinsic immune‐regulating capability of OCS‐robots. As mentioned above, the cellular immunity was triggered as the antigens were presented to CD8^+^ T cells through MHC I molecules while the humoral immunity was initiated as the antigen were presented to CD4^+^ T cells through MHC II molecules. In addition, the typical surface marker such as CD86 costimulatory molecules is essential for evaluating antigen presentations. Consequently, the expression level of CD86, MHC I and MHC II were determined for antigen cross‐presentation. Briefly, bone marrow dendritic cells (BMDCs) extracted from the hind limb bone of C57BL/6 mouse were treated by magnetic‐driven OCS and other controls. Compared with the control groups, a great deal of pseudopods was clearly visible on the surface of BMDCs in the OCS‐robots group, showing the activation and maturation of BMDCs (Figure S19A–D, Supporting Information). Furthermore, the expression level of these indicators was also detected by CLSM (**Figure** **6**A–C). The fluorescence intensity of CD86, MHC II and MHC I in OCS‐robots group were stronger than other controls (Figure S20, Supporting Information), indicating that the active motion of OCS‐robots induced more efficiency of antigens cross‐presentation. In addition to the visual observation, the expression level of the three indicators in different treatment was analyzed by flow cytometry. The BMDCs were first stained by CD11c, indicating the high purity of extraction (Figure S21, Supporting Information). It was found that the expression level of CD86 and MHC II were significantly higher in the group of OCS‐robots than other controls. More importantly, for antigens cross‐presentation, OCS‐robots triggered the highest level of MHC I expression, with 1.83‐fold greater than the free OVA group. In brief, OCS‐robots with controllable and remarkable moving performance effectively induce BMDCs maturation and ultimately improve antigens cross‐presentation, which is the prerequisite of robots for T cell's activation to kill cancer in vivo.

### 2.6 In Vivo Antitumor Effects of OCS‐Robot

**Figure 7:**
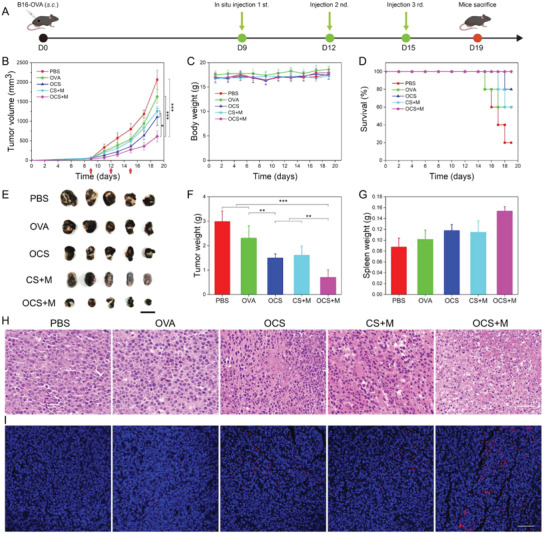
In vivo tumor therapeutic efficacy of OCS‐robot on B16‐OVA tumor‐bearing mice. A) Schematic illustration of the establishment of B16‐OVA tumor‐bearing mice and the treatments procedure in vivo. B) Tumor volume growth curves and C) body weight change curves of different groups. D) Survival rates of B16‐OVA tumor‐bearing mice in different groups. E) Photographs of excised tumors and F) the average tumor weight after different treatments. Scale bar, 2 cm. G) The average spleen weight after different treatments. H) H&E‐stained images of dissected tumors in different groups. Scale bar, 50 µm. I) Immunofluorescence images of CD8^+^ T cells (red) infiltration in section of tumor tissue. Scale bar, 100 µm. Data are represented as the mean ± SD (*n* = 5). Data analyzed by one way ANOVA test. *p < 0.05, **p < 0.01, and ***p < 0.001.

Encouraged by the enhanced cellular immunity of OCS‐robots, the therapeutic efficiency was further evaluated in the B16‐OVA tumor‐bearing mice model, which was employed to assess the specific immune response induced by OVA. After the volume of B16‐OVA tumors reached about 70 mm^3^ (on day 9), the mice were randomly divided into five groups (*n* = 5). The mice were treated with different formulations (PBS, OVA, OCS, CS+magnetic field and OCS + magnetic field, with OVA equivalent of 20 µg) by intratumor injection. The mice were placed in center of the homemade triaxial Helmholtz coil for magnetic actuation (Figure S22, Supporting Information). In order to further enhance the capture of antigen by DCs and subsequent maturation as well as cross‐presentation, the antigens were supposed to prolong the retention time and expose to DCs in vivo. Therefore, the fate of OVA/OCS was investigated by in vivo fluorescence images after injection for different times. Compared to the fast elimination of free antigen, the OCS carrier exhibited longer retention time even after 24 h (Figure S23, Supporting Information), which was facilitated for the internalization and cross‐presentation by DCs. Besides, the biodegradability of OCS‐robot was investigated by in vivo image with healthy mice. As shown in Figure S24, Supporting Information, the fluorescence signal of OCS‐robots was almost disappeared after 48 h, indicating the in vivo biodegradability of our design. In vivo MRI was also performed to evaluate the accumulation of OCS, and the *T*_2_ signal was dramatically increased after the injection (Figure S25, Supporting Information). The timeline of animal experiments was depicted in **Figure** **7**A, and the formulations were administered once every three days. The tumor volume and body weight of mice were monitored at two‐day intervals. Once the tumor volume reach 2000 mm,^[^ ^3^ ^]^ the mice ought to be sacrificed according to the guidelines of animal welfare. In this case, the mice were sacrificed and the tumor tissues and major organs were harvested on day 19th, as the tumor volume more than 2000 mm.^[^ ^3^ ^]^ It was clearly to see that the tumor volume in PBS and OVA groups displayed a remarkable increase (Figure 7B). Compared with these groups, OCS and CS‐robots groups exhibited the tumor inhibitory effect to some extent, but not satisfactory. Excitingly, the tumor volume was significantly inhibited after OCS‐robots treatment, which also demonstrated the specific killing effect compared with the CS‐robots without antigen OVA. According to the previous results, as the tumor microenvironment is typically immunosuppressive, the DCs is inactive in the acidic microenvironment so that the release of tumor antigen is not sufficient to activate the immune responses,^[^ ^12^ ^]^ which was also consistent with the results mentioned before. This was because the honeycomb OCS‐robot possessed the ability of neutralizing acidity and restoring viability of DCs. On the other hand, the OCS‐robots with active motion enhanced the phagocytosis by DCs and antigen cross‐presentation, which facilitated the activation of cellular immunity to fight against the tumor. The mice weights were also evaluated during the experiment and there was no significant difference in each group, indicating that there were hardly harmful to the mice body of different treatment (Figure 7C). Considering the mice tumor volume exceeding 2000 mm^3^ as dead, the survival rate of different treatment groups were compared (Figure 7D). All mice in the OCS + magnetic field group (OCS‐robots) survived in the 19 days treatment while the survival rate of PBS group remained 20% during the treatment, proving that OCS‐robot treatment could notably prolong the mice survival rate compared to the PBS. The same trend of tumor suppression rate was found in tumor photos and tumor weight (Figure 7E,F). Additionally, the group treated with OCS‐robots brought about the bigger spleen tissue compared to other control groups, demonstrating the awakening immune response (Figure 7G), which could be further demonstrated by more CD8^+^ T cells presenting in the spleen tissue (Figures S26 and S27B, Supporting Information). The results of histological evaluation using haematoxylin and eosin (H&E) staining also pointed to the therapeutic efficacy in various groups (Figure 7H). It was obvious that the OCS‐robot group experienced the worst cell necrosis and apoptosis of tumor tissue, which was consistent with the prominent therapeutic effect of the immunotherapy. As depicted in Figure 7I, it was obvious to see that an increase number of CD8^+^ T cells were infiltrated in the tumor tissues after OCS‐robots treatment. With the active motion of OCS robots, CD8^+^ T cells displayed a significant infiltration and distributed throughout the tumor tissue (Figure S27A, Supporting Information). These distinctive CD8^+^ T cell infiltration characteristics produced the various therapeutic outcomes.

**Figure 8:**
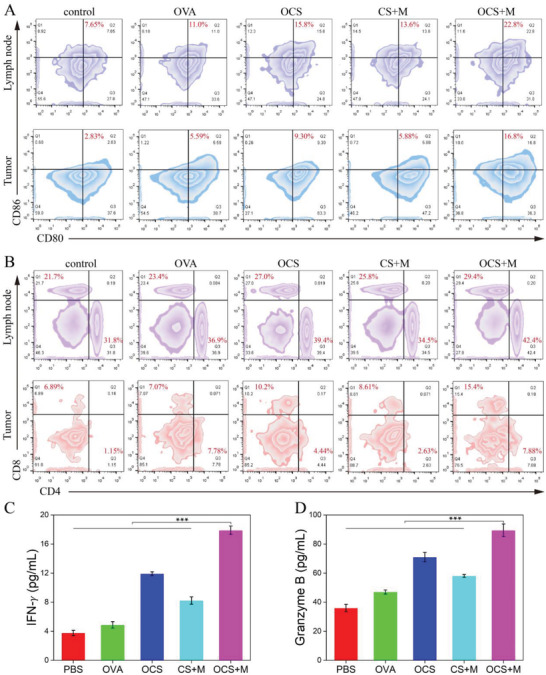
Effect of OCS‐robot on T cell responses on B16‐OVA tumor‐bearing mice model. Representative flow cytometry analysis of A) DC cells (CD11c^+^CD80^+^CD86^+^) and B) T cells (CD45^+^CD3^+^CD4^+^/CD8^+^) in lymph node and tumor tissue. C) IFN‐*γ* and D) granzyme B in the serum of mice by ELISA. Data are represented as the mean ± SD (*n* = 3) and analyzed by one way ANOVA test. ***p < 0.001.

In order to further investigate the immune responses induced by our OCS‐robots thoroughly, the lymph nodes and tumors were harvested and processed into single‐cell suspensions to evaluate DC cells (CD11c^+^CD80^+^CD86^+^) and T lymphocytes (CD45^+^CD3^+^CD4^+^/CD8^+^) by flow cytometry. The representative statistics of DCs in lymph node were depicted in **Figure** **8**A. It was demonstrated that the maturation of DCs (CD11c^+^CD80^+^CD86^+^) in the OCS treatment group (15.8%) was significantly higher than that in the PBS group (7.65%) and OVA group (11.0%), indicating the inherent immunoregulatory property and enhanced in situ antigen retention of OCS in vivo. However, the percentage of DC maturation was notably rose to 22.8% in the OCS‐robot treatment, indicating the active movement is possible to facilitate the efficient antigen delivery, recovery of cell viability and the following endocytosis of DCs. Compared with the CS‐robot without antigen OVA (13.6%), the OCS‐robot exhibited the specificity of activating the immune response. Subsequently, the T cells activation (CD45^+^CD3^+^CD4^+^/CD8^+^) in lymph node was also assessed by flow cytometry (Figure 8B). It can be clearly seen that the OCS‐robot treatment induced the highest percentages of CD4^+^ and CD8^+^ T cells among the other control groups, in agreement with the outcome of DC maturation, because DCs were homing to lymph nodes to activate T cells after maturation. Afterward, the tumor‐infiltrating immune cells were simultaneously analyzed. As shown in Figure 8A, a considerably higher proportion of mature DCs was revealed in tumor tissue after OCS‐robot treatment, indicating the intrinsic immunoregulatory property for efficient maturation of DCs. The same trend in the percentage of CD4^+^ and CD8^+^ T cells in tumor further supported the effective activation of the T cell‐mediated tumor immunotherapy (Figure 8B). These results demonstrated that the active motion of OCS‐robot had tremendous superiority in enhancing the immune response. As the cytotoxic T lymphocytes (CTLs) play a significant role in tumor eradication, the interferon‐gamma (IFN‐*γ*) and granzyme B cytokines in sera were determined by enzyme‐linked immunosorbent assay (ELISA). The secretory levels of IFN‐*γ* (Figure 8C) and granzyme B (Figure 8D) were considerably higher in OCS‐robots treatment compared to other groups, which demonstrated the best performance in terms of DC maturation, and CTL infiltration in tumor immunotherapy. Collectively, the favorable therapeutic effect was a result of active activation of immune system by OCS‐robot.

The major organs of heart, liver, spleen, lungs, and kidneys were harvested and stained by H&E for biosafety evaluation (Figure S28, Supporting Information). Compared with PBS group, no apparent pathological abnormalities were observed, confirming the negligible toxicity of OCS‐robots on the mice. Collectively, these OCS‐robots displayed tremendous advantage in tumor suppression, which indicated the potential application of OCS‐robots‐based system as a therapeutic cancer vaccine.

## 3 Conclusion

In summary, we demonstrated a wireless and effective strategy to activate the immune response by magnetically actuated dynamic vaccines delivery system. The developed CaCO_3_‐based nanorobot was constructed by integrating biocompatible SPIO and antigen OVA via a simple one‐pot synthesis, which was time‐saving, economical, biodegradable and suitable for large‐scale production. The OCS‐robots presented robust moving capability in a variety of physiological environment and controllable locomotion and navigation along the predesigned path under the rotating magnetic fields. Furthermore, the OCS‐robot was capable to transport a single cell or target a specific cell while intelligently avoided the obstacles in various motion behavior. With the ability of neutralizing acidity, this OCS‐robots with active motion could recover the viability of DCs which are inactive in the acidic tumor microenvironment, followed by enhance of cellular uptake. More importantly, this acid‐responsive capability was in favor of lysosome escape, which enhanced the antigen cross‐presentation and the subsequent cellular immunity. The in vivo results simultaneously proved the effect of immunotherapy by OCS‐robots. This work demonstrates a smart platform of employing dynamic vaccines delivery system (OCS‐robots) for active immunotherapy, which represents an outstanding therapeutic strategy for development of a new avenue in tumor treatment.

## 4 Experimental Section

Materials and Methods are available in the Supporting Information.

## Conflict of Interest

The authors declare no conflict of interest.

## Supporting information

Supporting Information

Supplemental Movie 1

Supplemental Movie 2

Supplemental Movie 3

Supplemental Movie 4

Supplemental Movie 5

Supplemental Movie 6

Supplemental Movie 7

Supplemental Movie 8

Supplemental Movie 9

## Data Availability

The data that support the findings of this study are available from the corresponding author upon reasonable request.
