# Supplementary material for: Magnetically Actuated Biodegradable Nanorobots for Active Immunotherapy
Source: Adv Sci (Weinh). 2023 Jun 29;10(25):2300540. doi: 10.1002/advs.202300540 (PMC10477856; doi:10.1002/advs.202300540)
Supplement: Supplementary file 1 — Supporting Information [file ADVS-10-2300540-s006.pdf]

ADVANCED SCIENCE
Open Access

Supporting Information

for *Adv. Sci.*, DOI 10.1002/advs.202300540

Magnetically Actuated Biodegradable Nanorobots for Active Immunotherapy

Yicheng Ye, Hao Tian, Jiamiao Jiang, Weichang Huang, Ruotian Zhang, Huaan Li, Lu Liu, Junbin Gao, Haixin Tan, Meihuan Liu, Fei Peng and Yingfeng Tu**

Supporting Information

Magnetically Actuated Biodegradable Nanorobots for Active Immunotherapy

Yicheng Ye†, Hao Tian†, Jiamiao Jiang, Weichang Huang, Ruotian Zhang, Huaan Li, Lu Liu, Junbin Gao, Haixin Tan, Meihuan Liu, Fei Peng and Yingfeng Tu**

1. Materials

Ferrous chloride tetrahydrate ($FeCl_2 \cdot 4H_2O$, 99%), ferric chloride hexahydrate ($FeCl_3 \cdot 6H_2O$, 99%), Calcium chloride dihydrate ($CaCl_2 \cdot 2H_2O$, 99%), Sodium carbonate anhydrous (Na_2CO_3, 99%), aqueous ammonia solution (25%-28%), Na_2HPO_4, NaH_2PO_4, Lactic acid (LA), rapamycin (RAPA), acetic acid buffer were purchased from Innochem. OVA were bought from Sigma-Aldrich (USA). Cy5-NHS was purchased from Shanghai Yuanye Biotechnology. Annexin V-FITC and PI was obtained from MultiSciences. Enhanced BCA Protein Assay Kit was obtained from Beyotime Biotechnology. Cell Counting Kit-8, Red Blood Cell Lysis Buffer and collagenase I were purchased from Solarbio Science & Technology Co., Ltd. (Beijing). Lyso-Tracker Green and DAPI were bought from Solarbio. Hoechst 33342 were bought from Coolaber Science & Technology. Cell Strainers (70μm) were obtained from SORFA Life Science. LC3-specific polyclonal antibody and Beclin 1 polyclonal antibody were bought from Cell Signaling Technology. GM-CSF was obtained from PeproTech. CD11c-FITC antibodies, CD80-APC antibodies, CD86-PE antibodies, MHC II-APC antibodies and MHC I-FITC antibodies, CD45-eFluor 506 antibodies, CD3-FITC antibodies, CD4-Percp Cy5.5 antibodies, CD8-PE antibodies were purchased from Biolegend. IFN-γ and Granzyme B Elisa kits were bought from BioLegend. The purified deionized water (18.2 MΩ) was used by the Milli-Q purification system (Millipore, USA).

The DC2.4 cells and B16-OVA cells were obtained from ATCC. These cell lines were cultivated at 37 °C with 5% carbon dioxide in DMEM media supplemented with 10% fatal bovine serum (FBS) and 1% penicillin-streptomycin solution.

2. Instruments

The structure of the prepared nanoparticles was characterized by a JEM 1400 Transmission Electron Microscope (TEM) with an acceleration voltage of 120 kV (JEOL, Japan). Scanning

electron microscope (SEM) images were captured by a Phenom emission scanning electron microscope with an acceleration voltage of 15 kV. The corresponding Energy dispersive X-ray spectroscopy (EDX) (installed on the Phenom FEI-SEM Pharos) was used for elemental analysis. Size measurements and Zeta potential measurements were performed on Malvern Zetasizer Nano ZS (Malvern, UK). Fourier-transformed infrared (FT-IR) spectra were acquired by a FT-IR spectrometer (Nicolet 380, Thermo). Powder X-ray diffraction (XRD) patterns were characterized by an X-ray powder diffractometer (D-MAX 2200, RIGAKU) using Cu Kα radiation with 2θ ranging in 10°- 80° at a temperature of 25 °C. The fluorescence intensity was measured with a Fluorescence spectrophotometer (SHIMADZU, Japan). The concentration of synthetic nanoparticles was measured by Inductivity Coupled Plasma-Mass Spectrometry (ICP-MS) (Thermo Fisher Scientific). The motion of OCS-robot was recorded by Ti2-A Inversion Fluorescence Microscope (Nikon, Japan) with a homemade three-dimensional Helmholtz coil system. Cell morphologies were captured by a Confocal laser scanning microscope (CLSM, Carl Zeiss, German). The *in vivo* imaging was acquired by In-Vivo FX PRO (BRUKER, Germany). The MR images were performed on a Philips Achieva 3.0T MRI system. The ultrasound images were obtained by an ultrasonographic imaging instrument (Aixplorer US system Supersonic Imagine, France) with a linear transducer with 4 to 15 MHz dimension of the probe. The blotted proteins of autophagy were analyzed by multifunctional imaging analysis system (FluorChem R, USA).

3. Synthesis of superparamagnetic iron oxide (SPIO)

SPIO were synthesized based on co-precipitation, which was facile, reproducible and suitable for large-scale production.[1] Briefly, 12.57 mmol $FeCl_2·4H_2O$ and 18.50 mmol $FeCl_3·6H_2O$ were dissolved in 100 mL of deionized water under continuous stirring (at 500 rpm). Then, 12 mL of aqueous ammonia solution was rapidly added. Instantly, a colloidal solution was formed. The precipitation was obtained by a magnet, and the pH value was brought to neutral by washing with deionized water. To create a homogeneous colloidal solution, the black colloid was dispersed in 100 mL of deionized water and subjected to an ultrasonic treatment for 6 h. The resultant SPIO with mass concentration of 20 mg/mL was kept at 4 °C.

4. Fabrication of OVA-$CaCO_3$-SPIO (OCS)

OCS was synthesized according to a previous report with some modifications.[2] The stock solutions of 0.5 M $CaCl_2$, 0.5 M Na_2CO_3 and 10 mg/mL OVA were firstly prepared. A mixture of $CaCl_2$ (100 μL), SPIO (100 μL) and OVA (4 mL) solution was obtained under moderate

stirring for 10 min. Afterwards, Na_2CO_3 (100 μL) was added dropwise into the solution under vigorous stirring for 20 min. OCS was collected by centrifugation, washed twice and redispersed in 5 mL DI water (1 mg/mL). The obtained particles were characterized by TEM, SEM, DLS, FTIR, TGA, XRD and magnetic hysteresis curve.

For the fluorescence labeling, Cy5-NHS (1mg/mL) was added in the OVA (10 mg/mL in PBS) solution under constant stirring for 4 h at room temperature under dark conditions. The synthesis of Cy5-OCS was similar to the above.

5. Motion of OCS-robots under magnetic field

The controllable motion of OCS-robots was actuated in the rotating magnetic field generated by a homemade three-dimensional Helmholtz coil system. OCS-robots with appropriate concentration were dispersed in different media (PBS, cell culture medium and diluted blood) in a cell counting chamber in order to minimize the drift producing phenomena. The samples were placed at the center of the Helmholtz coil device to acquire a homogeneous magnetic field and observed under a microscope (Nikon Ti2-A). Time-lapse images were recorded with ΔT = 100 ms. These image sequences were analyzed by ImageJ software with manual tracking plugin. The motion of OCS-robots was manipulated by changing the frequency and phase of the rotating magnetic field.

6. OCS neutralizing acidity and degradation behavior

Lactic acid was employed to prepare PBS with different pH values. The ability of OCS to neutralize acidity and degradation behavior of OCS at different pH conditions were firstly investigated. OCS with appropriate concentration was incubated with PBS with different pH values for 12 h. After that, the particles were collected and measured by DLS and TEM. Moreover, OCS with different concentrations were added into pH 6.5 buffer respectively and the resulting mixture were shaked for 24 h at 37 °C. Then, the supernatant was collected by centrifugation to detect the pH value.

7. Apoptosis of Dendritic Cells at different pH values

DCs were cultured in six-well plates and the culture medium was changed with different pH values (pH 6.5/ 7.0/ 7.4), followed by culturing for another 24 h. The resulting DCs were collected and stained with annexin V-FITC and PI for flow cytometry detection.

8. Cell viability of DCs with or without OCS at different pH values

DCs were seeded in 96-well plate with 10^4 cells per well. The medium was replaced by cell media with different pH values (pH 6.5, 6.8, 7.0, 7.2 and 7.4) with or without OCS, and the resulting cells were cultured for 24 h. After that, DCs were washed with PBS and incubated with medium containing 10 μL of Cell Counting Kit-8 for 2 h. The absorbance at 450 nm was detected and the cell viability of DCs was calculated.

9. Cellular uptake efficiency of DCs at different pH values

DCs were seeded in six-well plates and cultured with the medium of different pH values (pH 6.5/ 7.0/ 7.4). Cy5-OCS was then added and the resulting cells were further cultured for 24 h. After that, the cells were digested and detected for flow cytometry.

10. Cellular uptake efficiency of DCs to OCS at different time points

DCs were incubated with different formulations (Cy5-OVA or Cy5-OCS) in the glass confocal dish for different time points. The dish was positioned in the middle of the Helmholtz coil configuration for robot group (10 Hz, 10 min). For the static control, the Helmholtz coil setup was omitted. DCs were cultured for another 4, 6 and 12 h. After staining the nucleus with Hoechst 33342, the cells were observed by inverted fluorescence microscope.

11. Western Blotting

DCs were seeded into 6-well plates and treated with various formulations before using for western blot analysis. Briefly, DCs were incubated with PBS (negative control), OVA (6 μg/mL) and OCS (40 μg/mL) for 24 h. For the active motion, the cells treated with OCS groups was placed in the center of the Helmholtz coil configuration (10 Hz, 10 min). Besides, DCs were treated with rapamycin (2 μg/mL) for 5 h as a positive control. Cell deposits were obtained by digestion and centrifugation. RIPA lysis buffer was used to extract the total proteins from DCs. A BCA protein assay kit was used to determine the protein content. Expression of Beclin 1 and transformation of LC3-I (the cytosolic form) into LC3-II (membrane-bound lipidated form) are the important marker of autophagy, which were detected by the corresponding antibodies and immunoblotting. With glyceraldehyde-3-phosphate dehydrogenase (GAPDH) as an internal reference, the horseradish peroxidase (HRP)-enhanced chemiluminescence (ECL) detection method was utilized to stain the blotted proteins.

12. Lysosome co-localization and escape

To realize the cross-presentation of antigen, the intracellular fate of OCS was determined. Firstly, DCs were incubated with different formulations in the confocal dish. The magnetic field was applied for OCS-robot group. After incubation for different times, the nucleus was stained with Hoechst 33342 and the lysosome was stained with Lyso-tracker green, and the resulting cells were observed by Laser scanning confocal microscope.

13. MR Imaging and Ultrasound Imaging

For MR Imaging, different concentrations of OCS were suspended in DI water in 1 mL tubes. MRI image and value of OCS with different concentrations (0, 0.05, 0.1, 0.4, 0.6, 0.8, 1, 1.5 and 1.8 mg/mL) were obtained from a Philips Achieva 3.0T MRI system.

For the Ultrasound Imaging, equal volume of PBS or OCS (1 mg/mL) was injected in the hand-made water sac phantom filled with acetic acid buffer (pH 4.5) to mimic the lysosomes condition. The ultrasound images were acquired by an ultrasonographic imaging instrument.

14. Extraction and activation of bone marrow-derived cells (BMDCs)

BMDCs were extracted from the femurs of 6-week-old C57BL/6 female mice, and cultured for 6 days.[3] The cells were seeded in the confocal dishes (1×10^6 cells) and incubated with different concentrations of preparations (equivalent 5 μg/mL of OVA). The OCS-robot group was placed in the magnetic field (10 Hz, 10 min). After incubation for further 6 h, BMDCs were stained with anti-CD11c, anti-CD86, anti-MHC II and anti-MHC I for 20 min. The morphology of BMDCs was observed by Inverted fluorescence microscope while the expression levels of surface markers were analyzed using flow cytometry and CLSM.

15. Antitumor effect on B16-OVA tumor-bearing C57BL/6 mice model

Female C57BL/6 female mice (6-week-old) were purchased from Animal Experiment Center of Southern Medical University. All the animal experiments were carried out in accordance with the guideline approved by the Institutional Animal Care and Use Committee of Southern Medical University. To establish the tumor model, B16-OVA cells were collected and resuspended in the matrigel at a concentration of 3×10^{6} cells mL^{-1}. After that, 100 μL of the cells were injected into the right back of each mouse. The mice were randomly divided into five groups with five mice in each group after the average tumor volume reached about 70 mm^3. Afterward, the mice were intratumorally injected with 50 μL of PBS (PBS group), OVA (20 μg, OVA group), OCS (140 μg, OCS group), CS + magnetic field (140 μg, CS-robot group) and OCS + magnetic field (140 μg, OCS-robot group) for three times, respectively. The

magnetic field was performed for 30 min at 10 Hz once after the injection. The treatments were given every three days. Besides, the tumor volume and body weight of the mice were recorded every two days while the survival rate of the mice were monitored every day. After treating for 19 days, all the mice were sacrificed and the tumor tissues were imaged and weighted, followed by H&E staining and immunofluorescence analysis. In the meantime, the major organs tissues like heart, liver, spleen, lung and kidney were obtained for safety evaluation. The tumor volume was calculated using the formula: $V = \text{length} \times \text{width}^2/2$.

16. *In vivo* antitumor immune responses

To assess the immune response, the inguinal lymph nodes and tumors were obtained for the assessment of immune cells by flow cytometry.

For DCs maturation analysis, the inguinal lymph nodes of mice were collected and filtered to obtain the single-cell suspension. Then, the cell suspensions were stained by anti-CD11c-FITC, anti-CD80-PE and anti-CD86-APC antibodies for the activation and maturation of DCs by flow cytometry. Besides, the single-cell suspension of inguinal lymph nodes were also stained by anti-CD45-BV510, anti-CD3-FITC, anti-CD4-PerCP-Cy5.5 and anti-CD8-PE for the analysis of activation of T cells.

Tumors obtained from mice were digested by collagenase I and filtered through 70 μm filters to obtain the single-cell suspension. For DCs activation and maturation analysis, the cell suspensions were stained by anti-CD11c-FITC, anti-CD80-PE and anti-CD86-APC antibodies for flow cytometry investigation. For tumor-infiltrating T cells analysis, the cell suspensions were stained by anti-CD45-BV510, anti-CD3-FITC, anti-CD4-PerCP-Cy5.5 and anti-CD8-PE antibodies.

The Interferon-gamma (IFN-γ) and Granzyme B cytokines in sera were assessed by enzyme-linked immunosorbent assay (ELISA).

17. Statistical and Data Analysis

PRISM software 8.0 (Graph Pad Software) was employed. The mean ± SD were determined for all treatment groups. Student's t tests were used to compare two samples, and one-way analysis of variance (ANOVA) with post hoc testing were used to compare multiple samples. $P < 0.05$ was considered representative of a statistically significant difference between two groups.

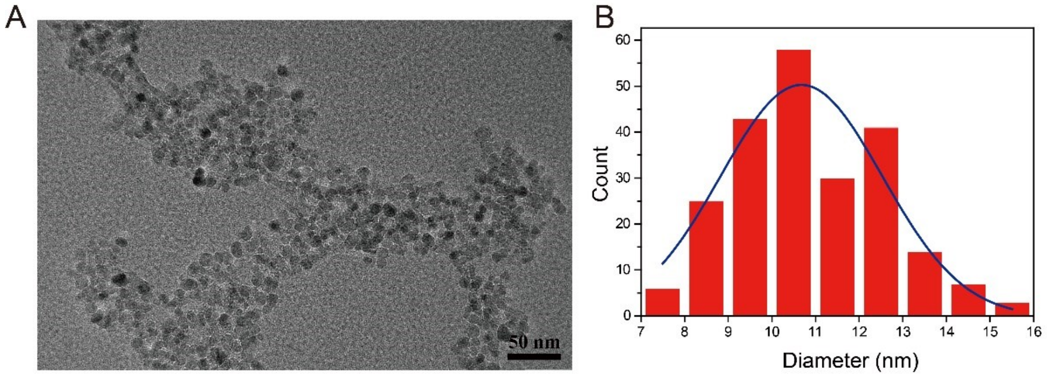

Figure S1. (A) TEM image of SPIO and (B) the corresponding size distribution (n = 227).

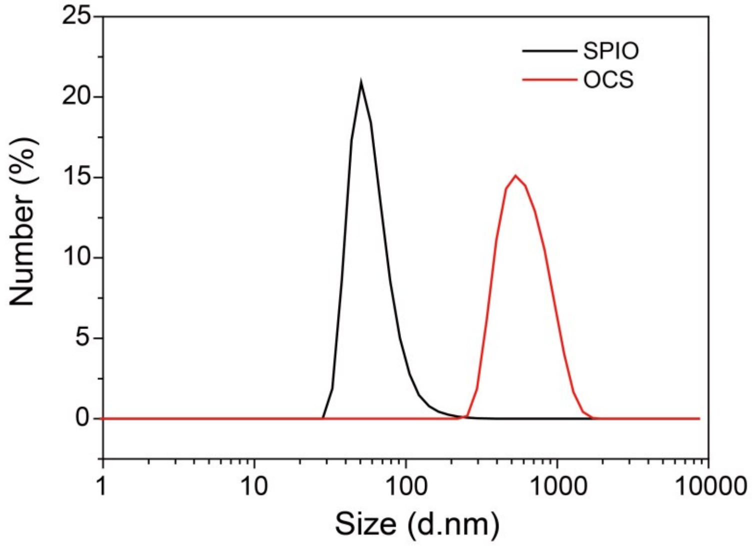

Figure S2. Hydrodynamic size distributions of SPIO and OCS.

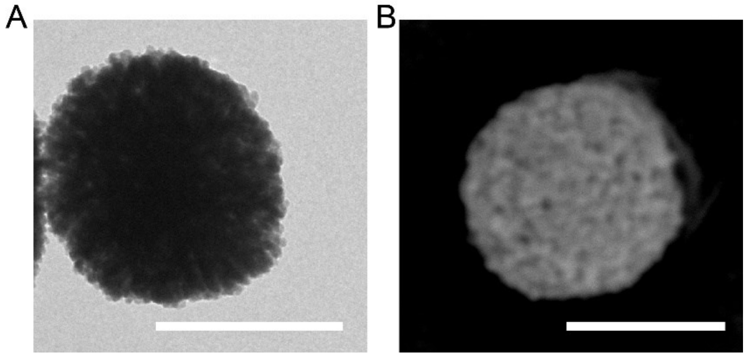

Figure S3. (A) TEM and (B) SEM image of the representative honeycomb OCS. Scale bar = 500 nm.

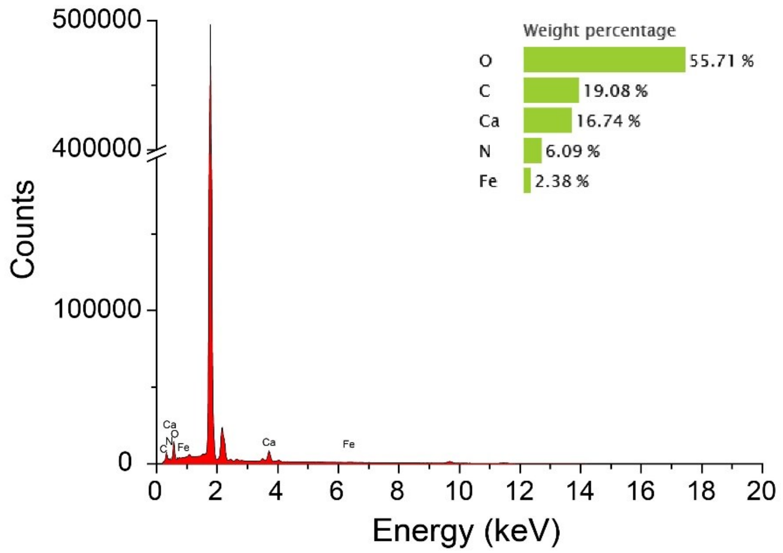

Figure S4. Energy dispersive X-ray (EDX) spectroscopy of OCS and the element weight percentage.

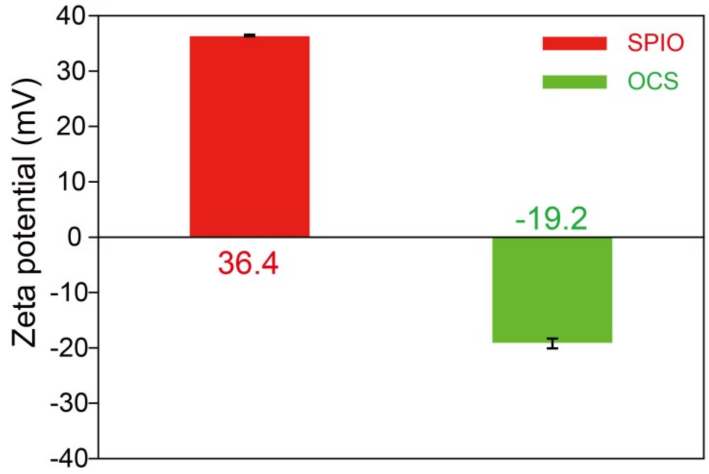

Figure S5. Zeta potential of SPIO and OCS.

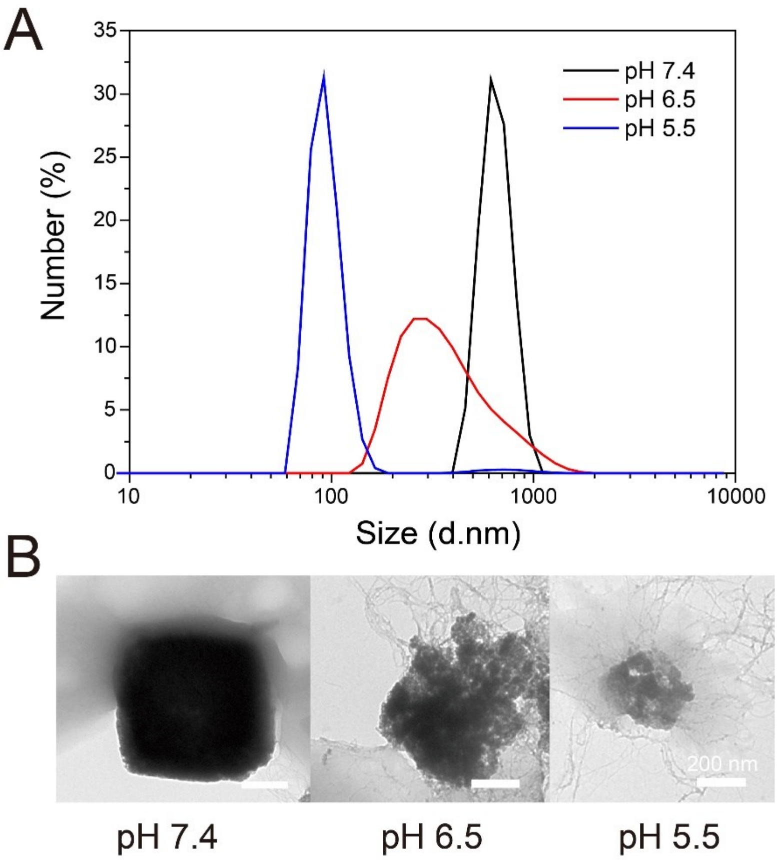

Figure S6. (A) Hydrodynamic sizes and (B) the corresponding TEM images of OCS under different pH values (pH 7.4, 6.5 and 5.5) for 12 h.

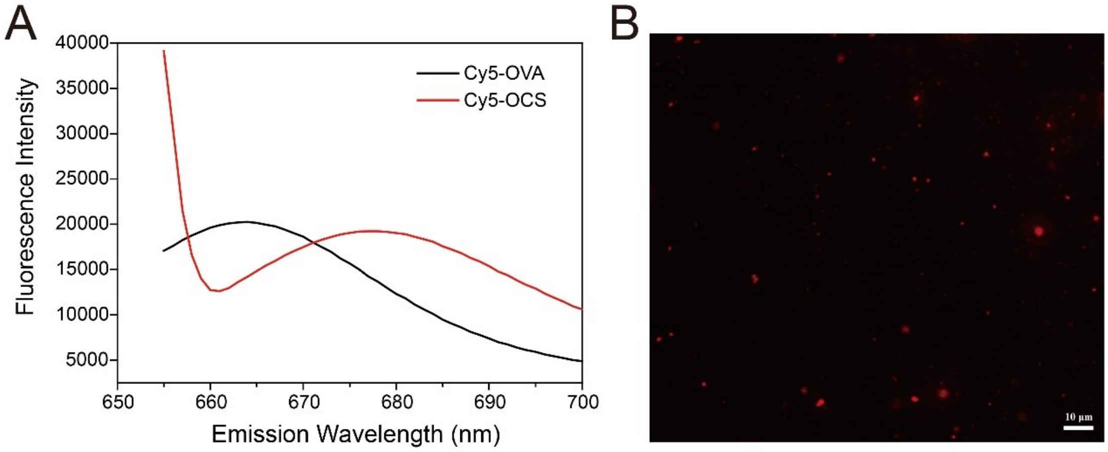

Figure S7. (A) Fluorescence emission spectra of Cy5-OVA and Cy5-OCS. (B) The fluorescence image of Cy5-OCS in DI water.

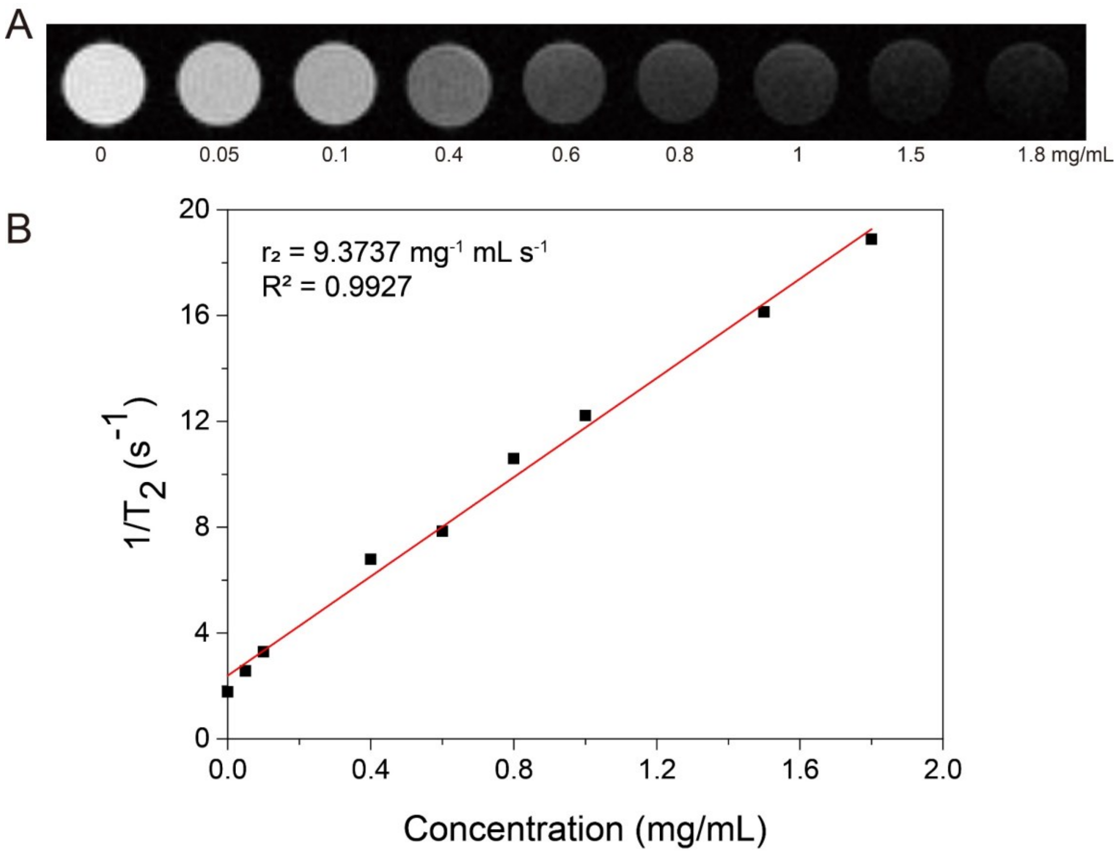

Figure S8. MRI image of OCS. (A) T_2-weight MRI images of OCS with different concentrations and (B) the corresponding r_2 value.

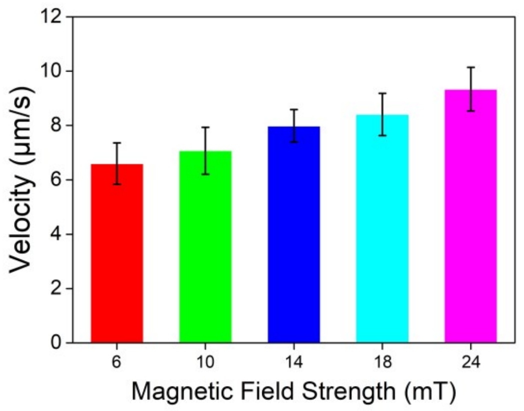

Figure S9. Velocity of OCS-robot with different magnetic field strength. Data are represented as the mean ± SD (n = 5).

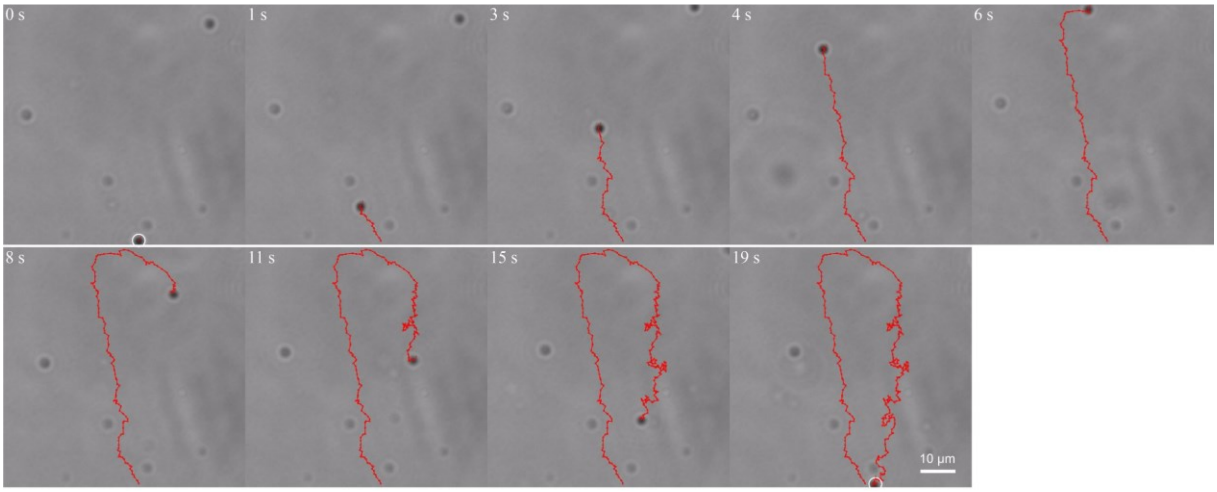

Figure S10. Video snapshots of a OCS-robot with circular motion behavior.

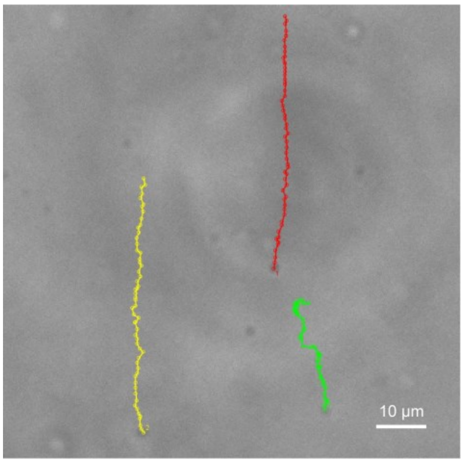

Figure S11. Time-lapse image of three OCS-robots after partial degradation in acidic microenvironment for 12 h.

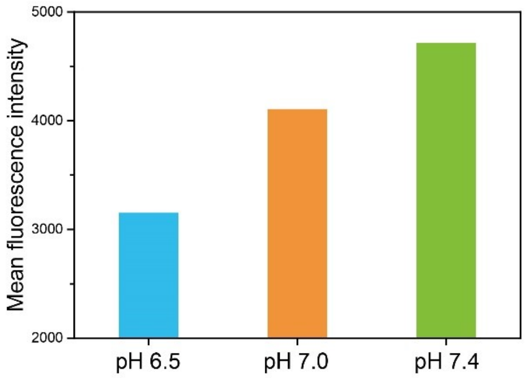

Figure S12. Mean fluorescence intensity of DCs uptake in different pH values of cell medium.

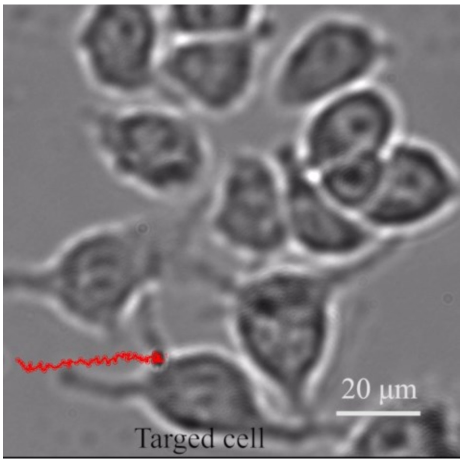

Figure S13. Time-lapse image of OCS-robot actively targeting the DCs.

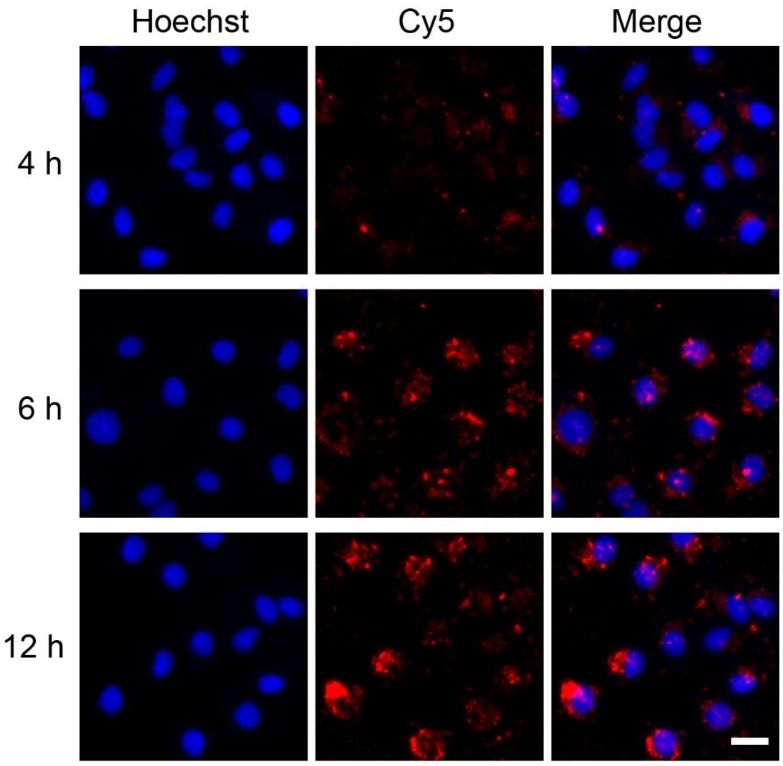

Figure S14. Effect of different incubation time on the uptake efficiency of DCs treated by OCS-robots. The nucleus were stained with Hoechst 33342 and OCS were labelled by Cy5. Scale bar = 25 μm. Magnetic field condition: 10 Hz, 10 min.

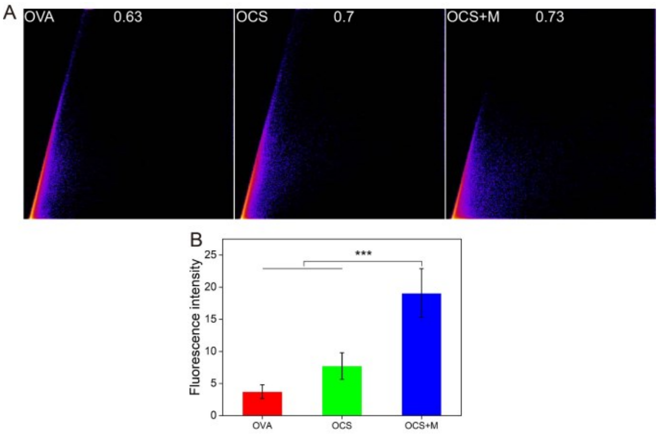

Figure S15. Quantitative data for the lysosome co-localization with different treatments. The Pearson's R value was 0.63, 0.7 and 0.73 in the group of OVA, OCS and OCS+M, respectively. Besides, the OCS-robots showed enhanced accumulation in the lysosome with the enhanced endocytosis. The fluorescence intensity of the OCS-robots was 2.47-fold higher than the OCS and 5.12-fold higher than the OVA. Data are represented as the mean ± SD (n = 3). Data are analyzed by one way ANOVA test. ***P < 0.001.

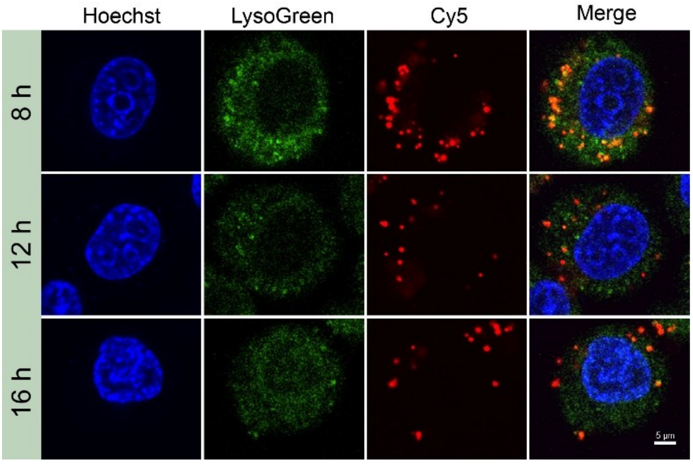

Figure S16. CLSM images of the lysosome co-localization and OCS-robots escape at different time intervals. The nucleus, lysosomes and OCS-robots were stained by Hoechst, Lysotracker-Green and Cy5, respectively. Magnetic field condition: 10 Hz, 10 min. The attenuated green fluorescence signal indicated the disrupted lysosome while the diffusive red fluorescence signal demonstrated that the dissolved particles had escaped from the lysosome to cytoplasm.

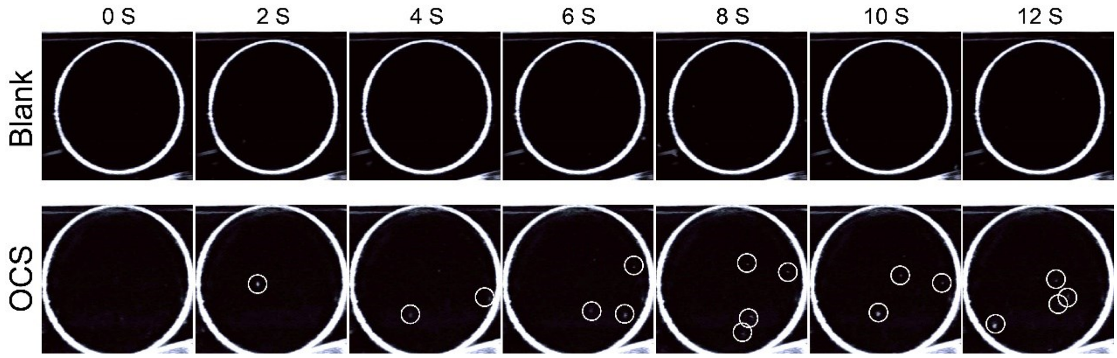

Figure S17. Time-dependent echogenic properties of blank and OCS groups in the acetic acid buffer (pH 4.5, lysosome-mimic medium).

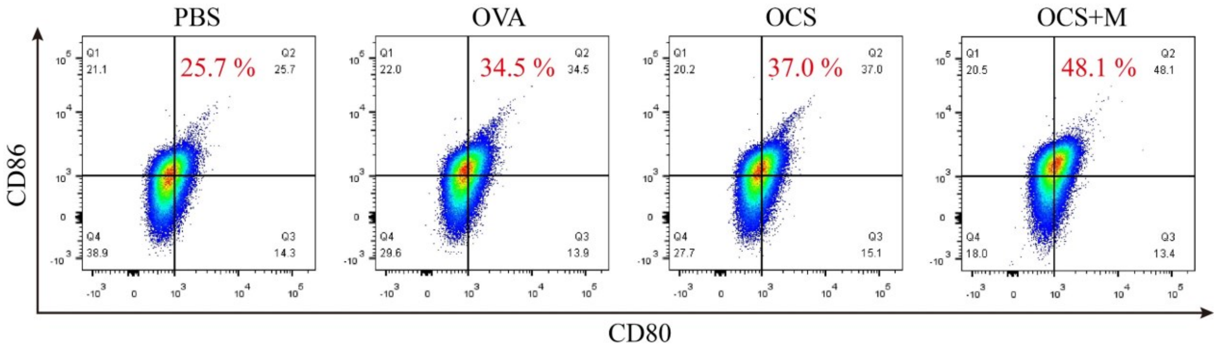

Figure S18. Activation and maturation of DC2.4 cells.

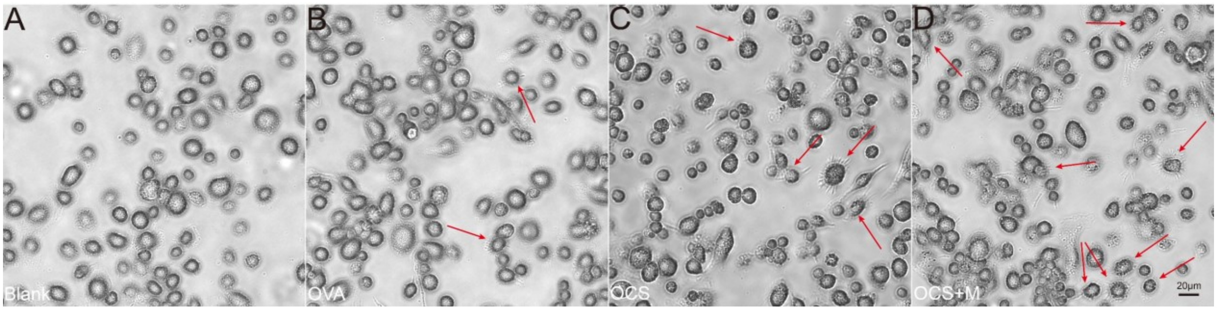

Figure S19. Activation and maturation of BMDCs extracted from mice. (A-D) Optical microscopic images of BMDCs after different treatments. The red arrows indicated the synapses of BMDCs.

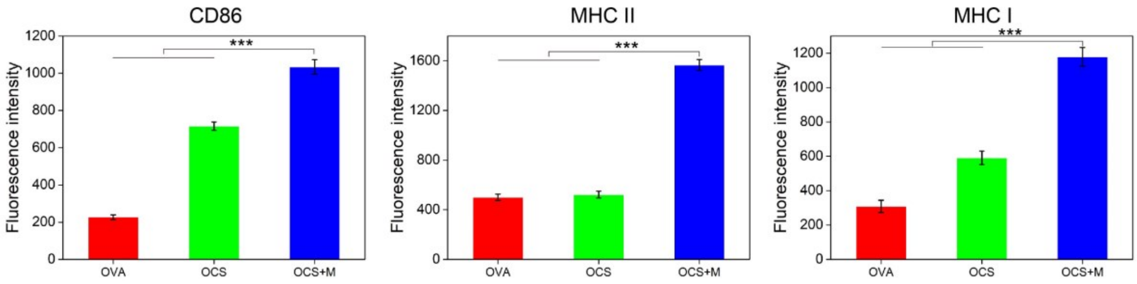

Figure S20. Quantitative data for the CLSM images of the expression of costimulatory marker CD86, MHC II and MHC I with different treatments. Data are analyzed by one way ANOVA test. ***P < 0.001.

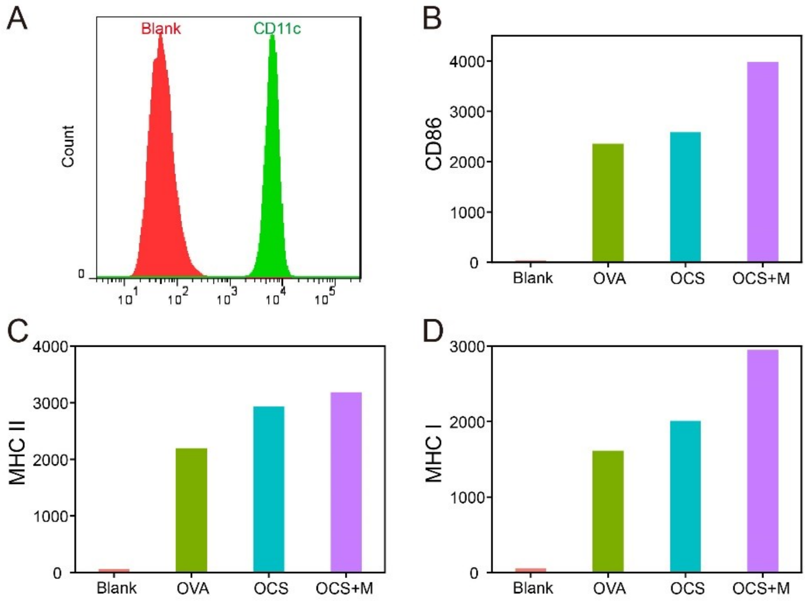

Figure S21. Activation and maturation of BMDCs detected by flow cytometry. (A) The extraction of BMDCs was confirmed by CD11c staining. The expression level of the surface marker CD86 (B), MHC II (C), and MHCI (D) after different treatments was analyzed by flow cytometry.

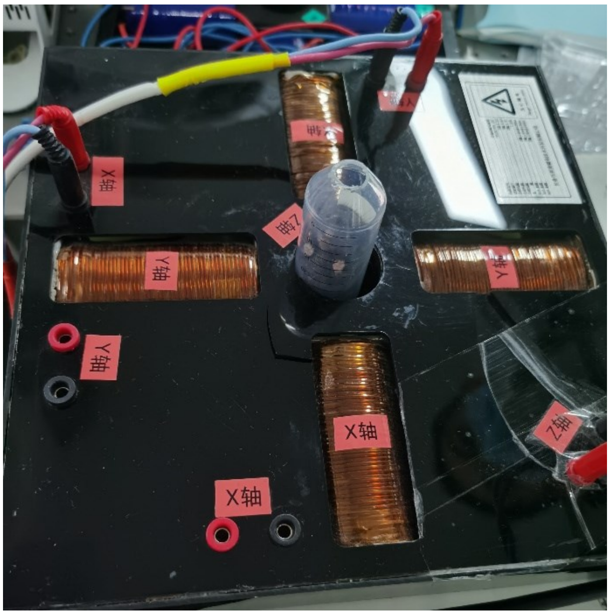

Figure S22. The mouse was placed in center of the homemade triaxial Helmholtz coil.

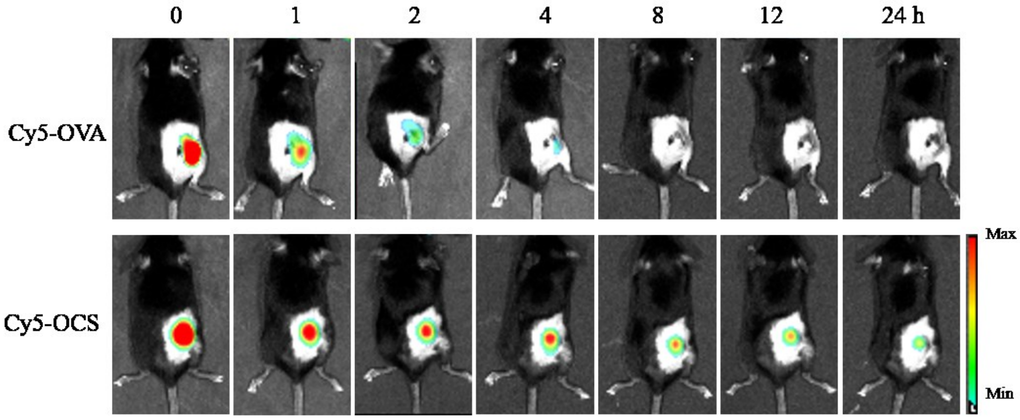

Figure S23. In situ antigen retention of free Cy5-OVA and Cy5-OCS in B16-OVA tumor-bearing mice with different time points.

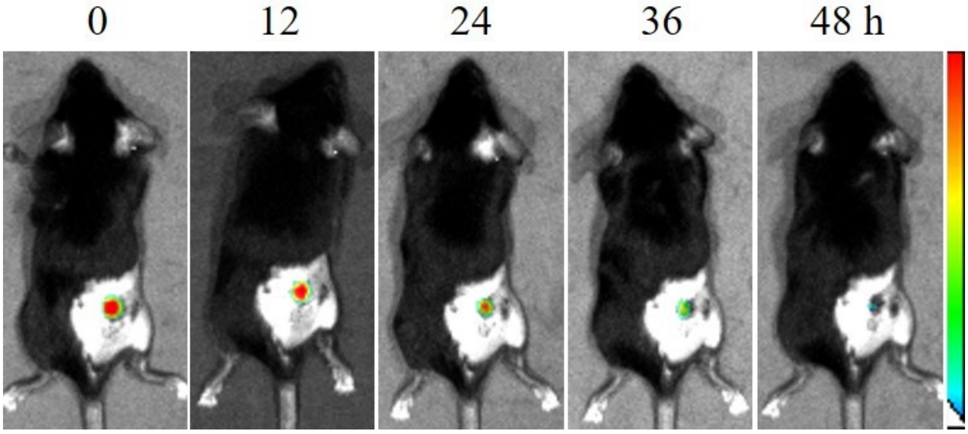

Figure S24. Biodegradability of OCS-robots *in vivo*.

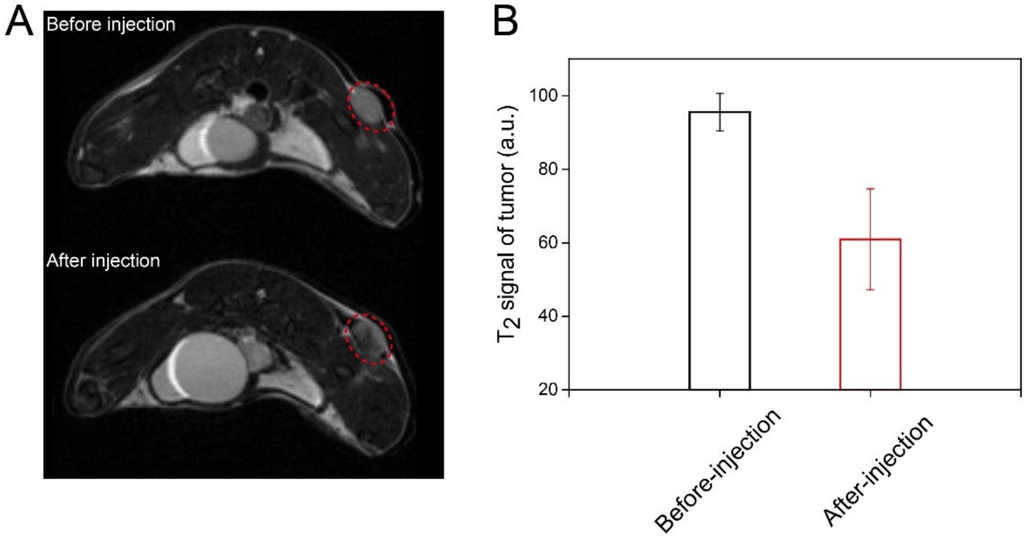

Figure S25. *In vivo* T_2 weighted MRI images of B16-OVA tumor-bearing mice before and after intratumoral injection of OCS. (A) The region in red dashed circles represented the tumor site. (B) The corresponding T_2 weighted signal of tumor region before and after injection (n=3).

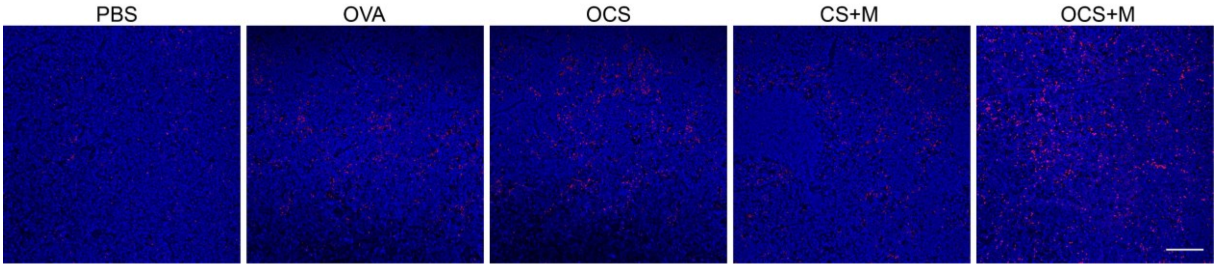

Figure S26. Immunofluorescence images of $CD8^+$ T cells (red) infiltration in the section of spleen tissue. Scale bar = 100 μm.

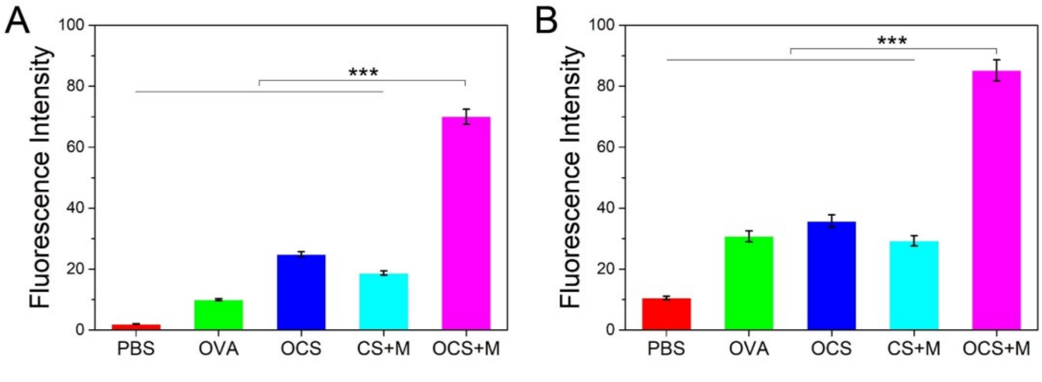

Figure S27. Quantitative data for the immunofluorescence images of $CD8^+$ T cells infiltration in the section of (A) tumor tissue and (B) spleen tissue. Data are analyzed by one way ANOVA test. ***$P < 0.001$.

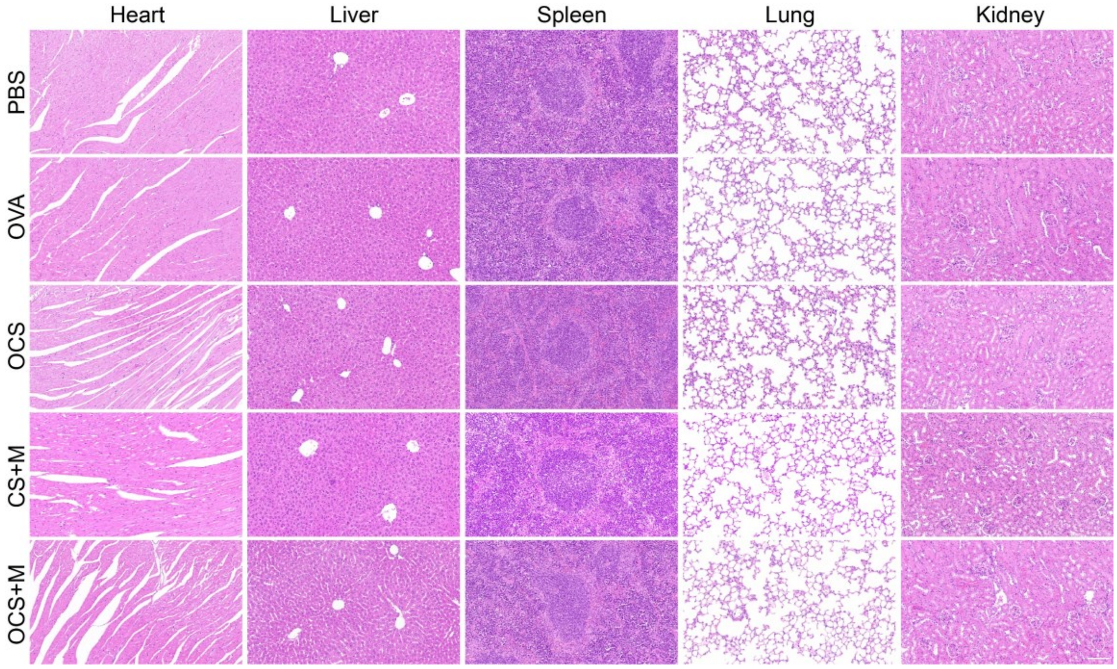

Figure S28. H&E stained images of heart, liver, spleen, lung, and kidney of B16-OVA tumor-bearing mice from different treatment groups. Scale bar = 100 μm.

References:
[1] N. Serov, A. Prilepskii, A. Sokolov, V. Vinogradov, *ChemNanoMat* **2019**, *5*, 1267-1271.
[2] S. Wang, D. Ni, H. Yue, N. Luo, X. Xi, Y. Wang, M. Shi, W. Wei, G. Ma, *Small* **2018**, *14*, 1704272.
[3] D. Zhu, C. Hu, F. Fan, Y. Qin, C. Huang, Z. Zhang, L. Lu, H. Wang, H. Sun, X. Leng, C. Wang, D. Kong, L. Zhang, *Biomaterials* **2019**, *206*, 25-40.

Supplementary Movies

Supplementary Movie 1. Magnetically actuated OCS-robot in PBS with different frequencies.

Supplementary Movie 2. Controllable magnetic propulsion of OCS-robot with the advance and return moving behavior.

Supplementary Movie 3. Controllable magnetic propulsion of OCS-robot with circular motion behavior.

Supplementary Movie 4. Controllable magnetic propulsion of OCS-robot performing a predefined track of "N".

Supplementary Movie 5. A swarm motion of OCS-robots.

Supplementary Movie 6. Motion of partially degraded OCS-robots.

Supplementary Movie 7. OCS-robot transporting a single cell.

Supplementary Movie 8. OCS-robot targeting a specific cell.

Supplementary Movie 9. The OCS-robot targeting the DC cell.
